# Recovery stories - helpful or unhelpful? A randomised controlled trial

**DOI:** 10.1186/2050-2974-2-S1-O50

**Published:** 2014-11-24

**Authors:** Lisa Dawson, Paul Rhodes, Barbara Mullan, Jane Miskovic, Stephen Touyz

**Affiliations:** 1 The University of Sydney, Sydney, Australia; 2 Curtin University, Perth, Australia; 3 The Children's Hopsital, Westmead, Sydney, Australia

## 

Low motivation to change and low self-efficacy have been associated with poorer outcome in anorexia nervosa (AN). There is evidence to suggest that sharing personal accounts of successful recovery with patients might improve motivation as well as helplessness and hopelessness associated with recovery, providing an important resource for sufferers. However, no research to date has explored the helpfulness or unhelpfulness of recovery narratives, despite many patients accessing such stories. The aim of the current study was to determine the efficacy of recovery narratives as a means of improving motivation and self-efficacy, using a randomised controlled trial design. The primary outcome variable was change in motivation as measured by intentions to recover from AN and stage of change. More than fifty individuals with AN and subclinical AN participated in this online study. Participants were randomised to either receive recovery stories or to a wait-list controlled group. After completing base-line measures, participants read five short stories about recovery from AN, and completed post-intervention measures two weeks later. Preliminary findings revealed that participants self-reported varying levels of usefulness. Full results regarding the effectiveness of the intervention as a means of improving motivation and self-efficacy are presented and clinical and research implications discussed.

This abstract was presented in the **Learning from Consumers** stream of the 2014 ANZAED Conference.

